# F203. A META-ANALYSIS OF MINOR PHYSICAL ANOMALIES IN FIRST-DEGREE UNAFFECTED RELATIVES OF PATIENTS WITH SCHIZOPHRENIA

**DOI:** 10.1093/schbul/sby017.734

**Published:** 2018-04-01

**Authors:** Ozge Akgul, Emre Bora, Berna Binnur Akdede, Köksal Alptekin

**Affiliations:** 1 Dokuz Eylul University; 2 Dokuz Eylül University School of Medicine

## Abstract

**Background:**

Neurodevelopmental abnormalities are common in schizophrenia. Minor physical anomalies (MPAs) are associated with abnormalities in neural development. Previous studies clearly demonstrated that MPAs are significantly increased in schizophrenia. However, the available evidence in unaffected relatives of patients with schizophrenia is contradictory.

**Methods:**

A literature search was conducted between 1 JAN 1980 and SEP 2017 in PUBMED and SCOPUS. Random-effects model was used. Heterogeneity was tested with Q test and I2. The meta-analysis was conducted using OpenMetaAnalyst software.

**Results:**

16 studies were included in the meta-analysis. MPAs were significantly more common unaffected first-degree relatives of patients with schizophrenia (d=0.56, CI=0.40–0.73, p<0.001). There was a significant heterogeneity in distribution of effect sizes (Q=42.2, p<0.001). The level of this heterogeneity was medium in range (I2=64 %). In meta-regression analyses, demographic variables were not significantly related with magnitude of the effect size.

**Discussion:**

MPAs are associated with risk of schizophrenia. However, the level of heterogeneity suggests that risk of psychosis is associated with neurodevelopmental abnormalities in some but not all individuals. Findings also emphasize that resilience factors might be protecting many neurodevelopmentally impaired relatives of schizophrenia against having a full-blown psychotic disorder.

